# Tubo-Ovarian Abscess With Bacteremia in a Patient With an Intrauterine Contraceptive Device

**DOI:** 10.7759/cureus.103964

**Published:** 2026-02-20

**Authors:** Malika Parchment, Mihir Waykar, Shannon Schellhammer, Steve Carlan

**Affiliations:** 1 Obstetrics and Gynecology, Orlando Regional Medical Center, Orlando, USA

**Keywords:** acute pelvic pain, gram-negative bacteremia, intrauterine devices (iud), pelvic inflammatory disease (pid), toa

## Abstract

A tubo-ovarian abscess (TOA) is a complication of pelvic inflammatory disease (PID) resulting from an ascending infection of the upper genital tract. It is characterized by an inflammatory mass involving the fallopian tube, ovary, and sometimes adjacent pelvic organs. It is diagnosed through clinical evaluation and imaging, and initially treated with broad-spectrum antibiotics. Despite conservative management, surgical intervention may be necessary if there is a leak or rupture of the TOA or if the patient experiences chronic pain. Rarely, a TOA can cause septicemia. Insertion of an IUD in a patient with a history of PID is contraindicated.

A 41-year-old, gravida 2, para 1011 (G2P1011) woman presented with a TOA complicated by *Escherichia coli *bacteremia. She experienced acute abdominal pain, fever, nausea, and vomiting. An intrauterine contraceptive device (IUD) had been inserted one year prior to her presentation. She had a history of PID 20 years earlier. Pelvic computed tomography (CT) and transvaginal ultrasound showed findings consistent with TOA. After multiple courses of inpatient and outpatient antibiotics, she eventually underwent diagnostic laparoscopy, bilateral salpingectomy, and lysis of adhesions. During surgery, she was found to have extensive adhesions, bilateral salpingitis, and a right pyosalpinx suspicious for rupture or leaking.

The timing of her IUD insertion and the onset of active pelvic infection was likely not coincidental. TOA complicated by *E. coli* bacteremia and suspected rupture or leakage presents a rare but serious clinical scenario. This case highlights the importance of early recognition of systemic infection signs, careful use of imaging and culture data, and prompt surgical intervention when conservative management fails.

## Introduction

Tubo-ovarian abscess (TOA) is a serious complication of pelvic inflammatory disease (PID), characterized by an inflammatory mass involving the fallopian tube, ovary, and occasionally adjacent pelvic organs. It mainly affects sexually active women of reproductive age. PID is an infection of the upper female reproductive tract, usually caused by ascending sexually transmitted or vaginal bacteria, and may be polymicrobial [1]. Approximately 15%-35% of hospitalized women treated for acute PID will develop a TOA [1]. Rupture or leaking of a TOA is a life-threatening emergency, causing peritonitis and sepsis, and requires urgent surgical intervention. About 15% of TOA cases result in rupture, with mortality rates reaching up to 4% despite modern therapies [2]. The association between an intrauterine contraceptive device (IUD) and TOA is context-dependent, rather than universally contraindicated. We present the case of a 41-year-old woman with an IUD in place for one year and a history of PID 20 years prior to admission, who showed signs, symptoms, and imaging consistent with a TOA and *Escherichia coli* bacteremia in the setting of failed medical management. A TOA complicated by *E. coli* bacteremia and suspected rupture despite partial response to antibiotics is extremely rare.

## Case presentation

A 41-year-old woman, gravida 2, para 1011 (G2P1011), presented with a 24-hour history of fever, abdominal pain, chills, diarrhea, nausea, and vomiting. Her menstrual period began the day before presentation, and she denied any dyspareunia or vaginal discharge. Her gynecologic history included a previous episode of TOA and chlamydia infection approximately 20 years ago. At that time, she was treated with both antibiotics and surgical intervention. The surgery performed was described as "diagnostic" and did not involve the removal of the fallopian tube or ovary.

**Figure 1:**
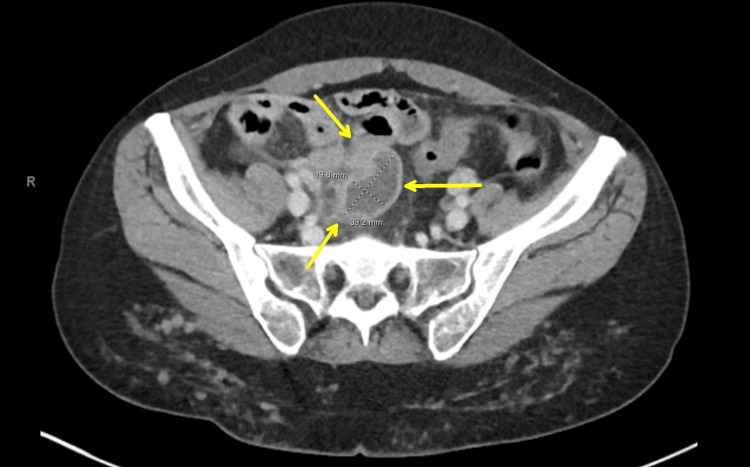
Computed tomography scan of the abdomen and pelvis (CTAP) showing a 3.9 x 1.98 x 3.7 cm rim-enhancing complex fluid collection in the right adnexa (yellow arrows).

She had a previous surgical history of an appendectomy. She had a levonorgestrel (IUD) inserted the year before admission. Upon admission, her body mass index (BMI) was 22.93 kg/m², temperature 98.5°F, heart rate 100 bpm, respiratory rate 16, and oxygen saturation (SpO₂) 99%. Her abdomen was soft but tender to palpation in the right lower quadrant (RLQ). Guarding was present, but there was no rebound tenderness or signs of peritonitis or acute abdomen. She experienced cervical motion tenderness and rectal discomfort on bimanual exam. Due to guarding, no distinct mass was detectable. No mucopurulent cervical discharge was observed. Pregnancy, chlamydia, gonorrhea, HIV, syphilis, hepatitis B, and hepatitis C tests were performed, all of which were negative. Blood and urine cultures were obtained. The complete blood count (CBC) showed a white blood cell (WBC) count of 18,900 cells/µL (normal range 4,500-11,000 cells/µL). A computed tomography scan of the abdomen and pelvis (CTAP) revealed a 3.9 x 1.98 x 3.7 cm rim-enhancing fluid collection in the right adnexa (Figure 1).

**Figure 2:**
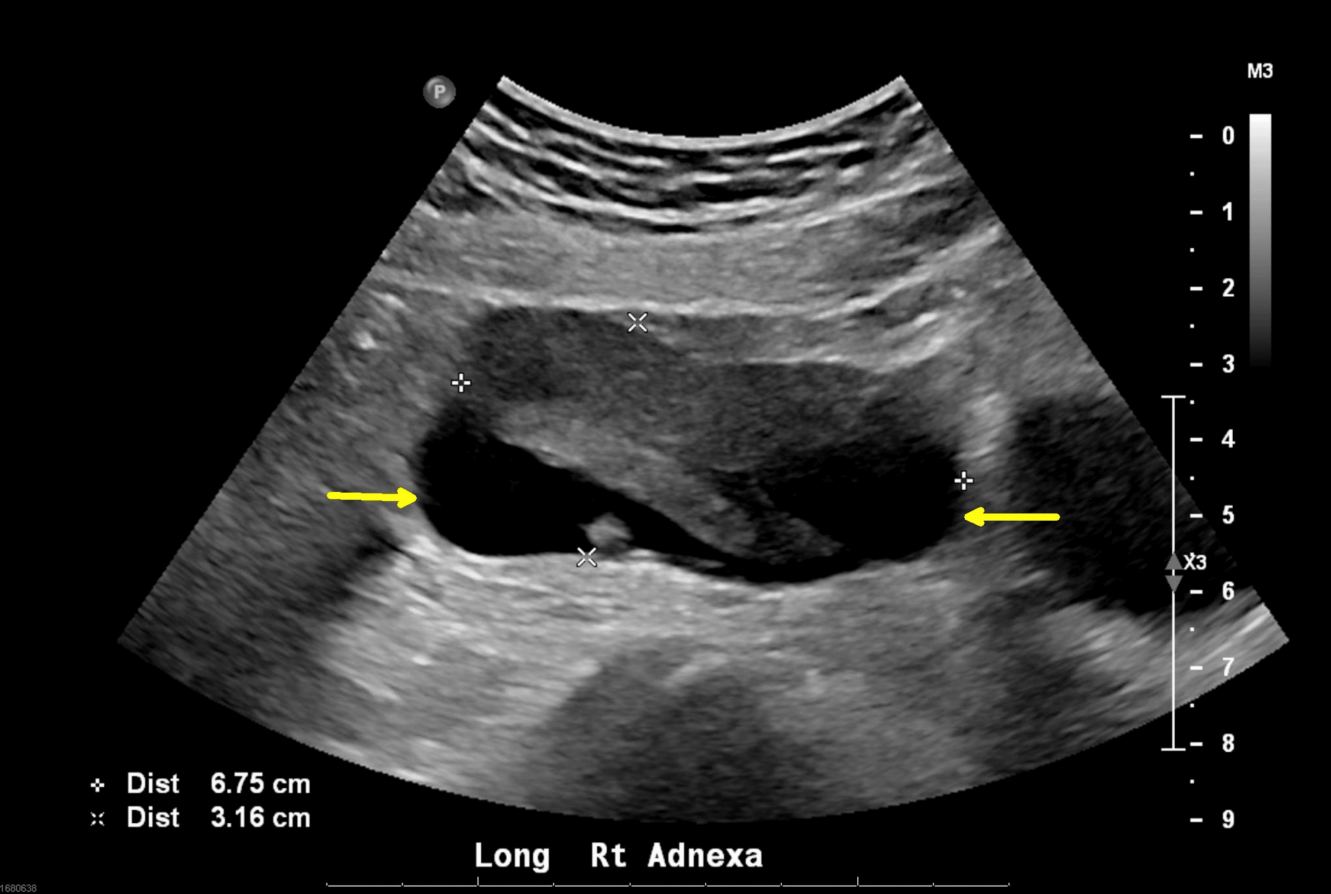
Pelvic ultrasound showing a 6.7 x 3.9 x 3.1 cm complex mass in the right adnexa with a tubular fluid component (between the yellow arrows).

Pelvic ultrasound showed a 6.7 x 3.9 x 3.1 cm heterogeneous complex in the right adnexa with a tubular fluid component. No free fluid in the abdomen was noted (Figure 2).

Upon admission, she was started on empiric ceftriaxone, metronidazole, and doxycycline for presumed pelvic inflammatory disease and TOA, and interventional radiology (IR) was consulted for evaluation for drainage. Initially, her vital signs were stable, then progressed to intermittent tachycardia and fever (Table 1).

**Table 1:** Serial data of the patient

Data on first admission	Normal range	Day 1	Day 2	Day 3	Day 4	Day 5	Day 6	Day 7	Day 8	Day 9	Day 10	Day 63
Hemoglobin (g/dL)	Women: -12.3 to 15.3	13.1	11.6	10.9	10.1	9.8	10.8	10.5	10.9	11.1	11.8	11.7
White blood cell (cells/µL)	4,500-11,000	18,900	21,900	17,000	11,700	8,400	8,700	8,100	9,300	8,100	8,600	8,100
Platelet (x10^9^/L)	100,000-400,000	203,000	185,000	19,000	193,000	223,000	272,000	288,000	303,000	337,000	379,000	294,000
Peripheral blood cultures			E. coli									
Urine culture			Negative									
Blood pressure (mmHg)	120/80	121/49	114/56	122/59	114/50	132/63	132/63	118/50	122/59	125/64	102/55	
Oxygen saturation (%)	95-100	99	91	96	89	93	94	95	97	98	99	
Heart rate (bpm)	60-100	101	126	100	74	74	71	82	78	78	79	
Temperature (°F)		98.4	101.6	99.1	98.2	98.6	98.1	99.3	98	98.2	98.8	

**Figure 3:**
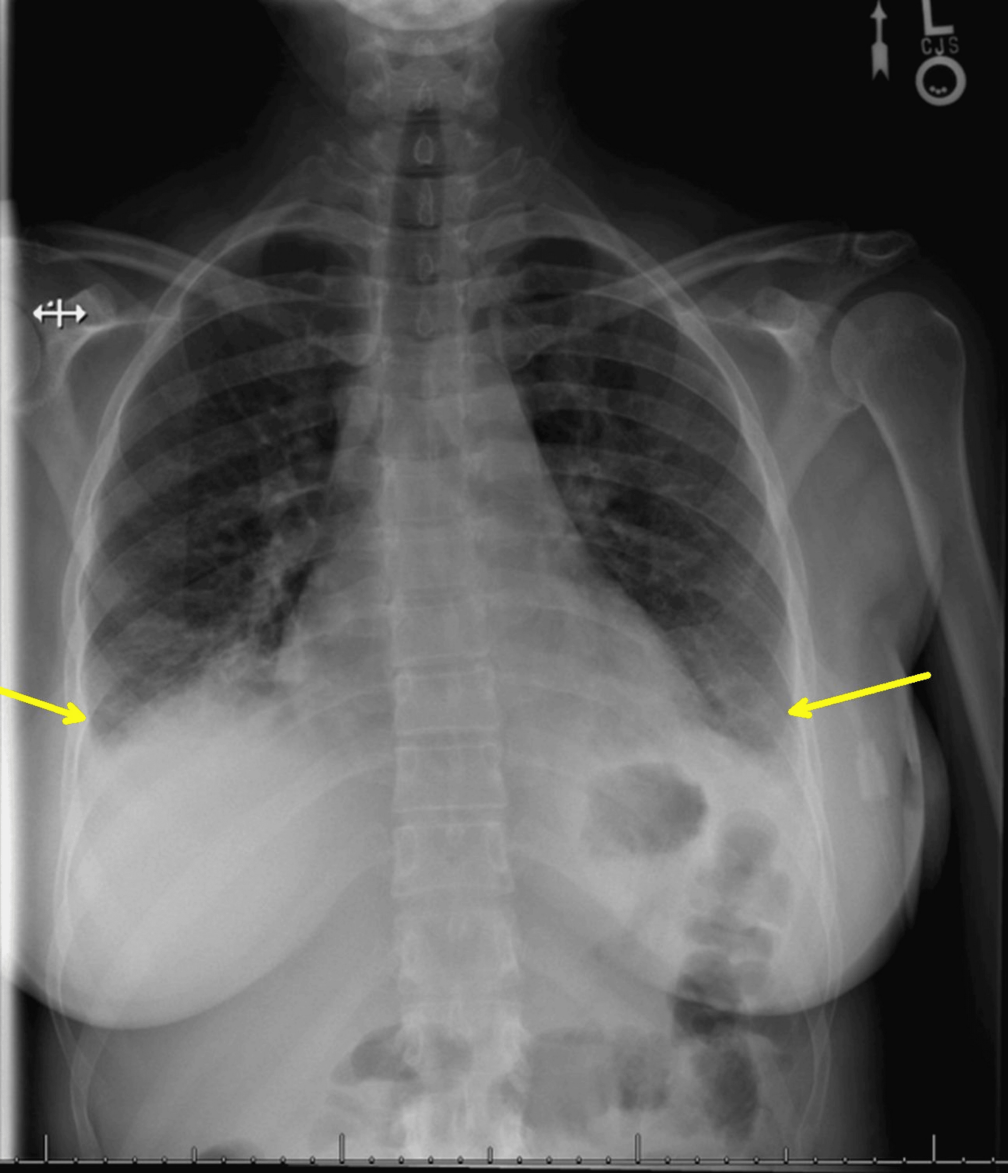
Chest radiograph demonstrating dependent pleural effusions (yellow arrows) and patchy bilateral alveolar pulmonary opacities.

Her maximum temperature reached 101.6°F, with a peak WBC of 21.9 on hospital day 2. Infectious disease was consulted on hospital day 2 after blood cultures showed gram-negative bacilli and *E. coli* on film array. Consequently, ceftriaxone dosing was increased from 1 g to 2 g every 24 hours per their recommendations. During this time, the patient reported an improvement in her abdominal pain and developed an appetite but required higher doses of intravenous pain medications for adequate relief until hospital day 4. IR deemed the suspected TOA unsafe for drainage due to its location. On hospital day 4, she began complaining of chest pain, continued abdominal pain, and oxygen desaturations to 89% on pulse oximetry, requiring supplemental oxygen via nasal cannula. Her workup revealed dependent pleural effusions on chest radiograph (Figure 3), and internal medicine recommended incentive spirometry and intravenous furosemide for presumed iatrogenic volume overload or an evolving systemic inflammatory response from infection.

There was no evidence of pulmonary embolism or congestive heart failure due to pump malfunction. The patient's pain and chest discomfort improved within 24 hours. Her leukocytosis continued to trend downward toward normal by hospital day 5, and her oxygen saturation returned to the high 90s. Before discharge, she developed mild transient transaminitis secondary to ceftriaxone, and infectious disease specialists recommended replacing it with ampicillin-sulbactam. She was discharged home on hospital day 10, when her liver enzymes decreased, and her abdominal pain had significantly improved.

**Figure 4:**
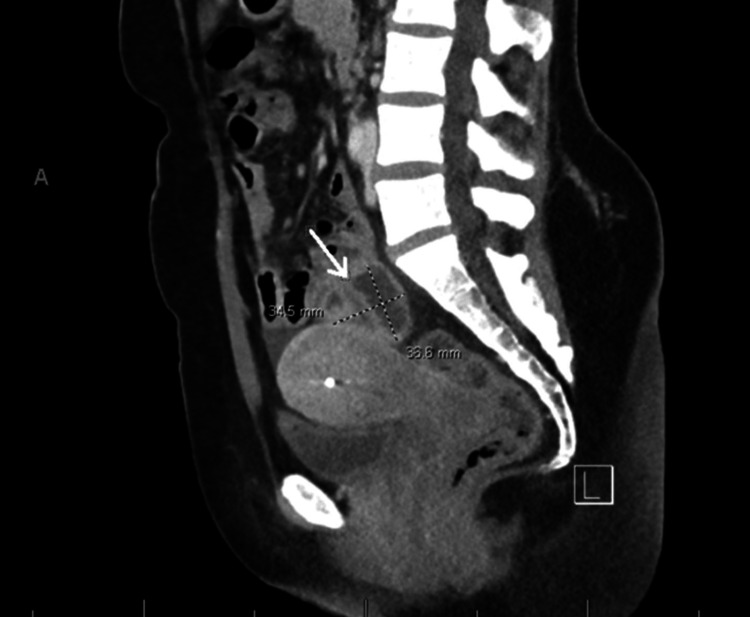
Longitudinal CT of the abdomen and pelvis demonstrating a large retrouterine mass (arrow) measuring 34.5 × 36.6 cm. The bright white spot within the uterus represents an intrauterine device (IUD).

She was prescribed a seven-day course of doxycycline and metronidazole, with instructions to follow up closely with infectious disease and gynecology, and to repeat CTAP in two weeks. She visited the gynecology three weeks after discharge and reported improvement in all symptoms except for intermittent abdominal pain. The repeat CTAP showed findings consistent with prior imaging, revealing a mixed-density pelvic mass measuring 34.5 by 36.6 mm (Figure 4).

**Figure 5:**
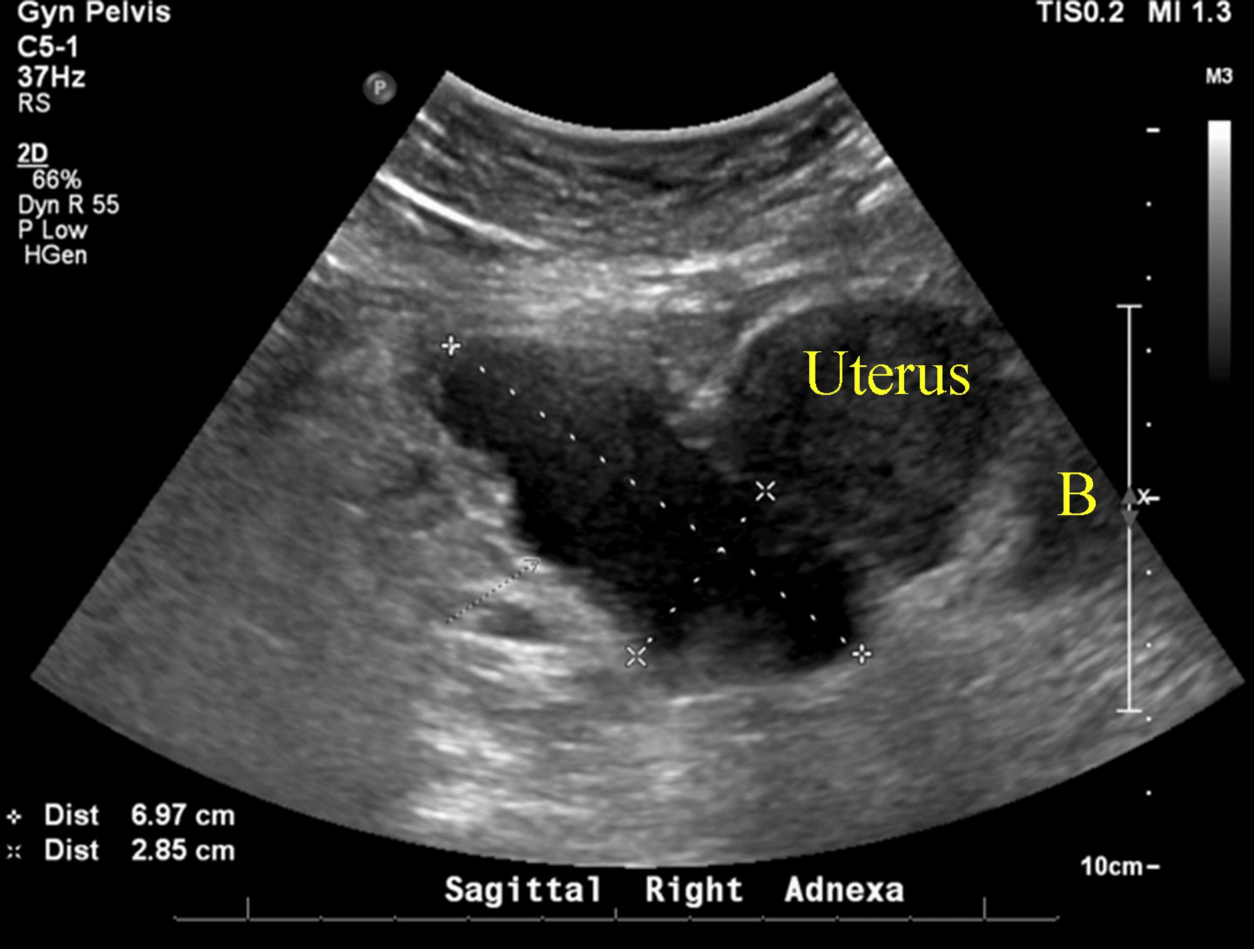
Transvaginal ultrasound demonstrating a tubo-ovarian abscess (TOA) measuring 6.9 x 2.8 cm. B: bladder.

At this point, the infectious disease recommended oral amoxicillin-clavulanate and suggested additional repeat imaging after completing the two-week antibiotic course. During her gynecology follow-up two weeks later, the patient was asymptomatic, and transvaginal ultrasound revealed a TOA measuring 6.9 x 2.8 cm (Figure 5).

Pelvic examination found the inflammatory mass palpable, though with some difficulty due to voluntary guarding.

**Figure 6:**
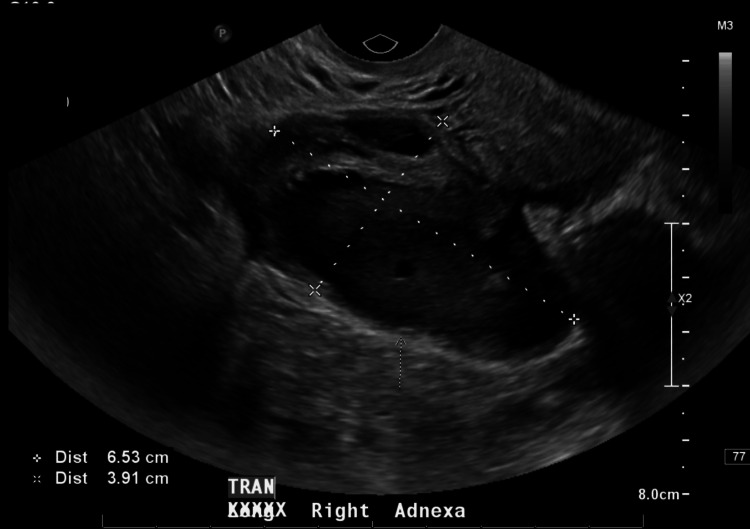
Transvaginal ultrasound nine weeks after the discharge, revealing a stable tubo-ovarian complex measuring 6.5 x 3.9 cm. No ascites were present.

Around nine weeks after discharge, she returned to the office with a recurrence of abdominal pain and nausea, although she denied having a fever or chills. On examination, she exhibited tenderness in the RLQ, purulent vaginal discharge, and positive cervical motion tenderness. In the office, a vaginitis panel and culture were collected, and she was sent to the emergency department (ED) for further evaluation with a CBC and transvaginal ultrasound. The CBC was within normal limits, and the transvaginal ultrasound showed a stable tubo-ovarian complex measuring 6.5 x 3.9 cm (Figure 6) and a possible malpositioned IUD into the left uterine side wall.

**Figure 7:**
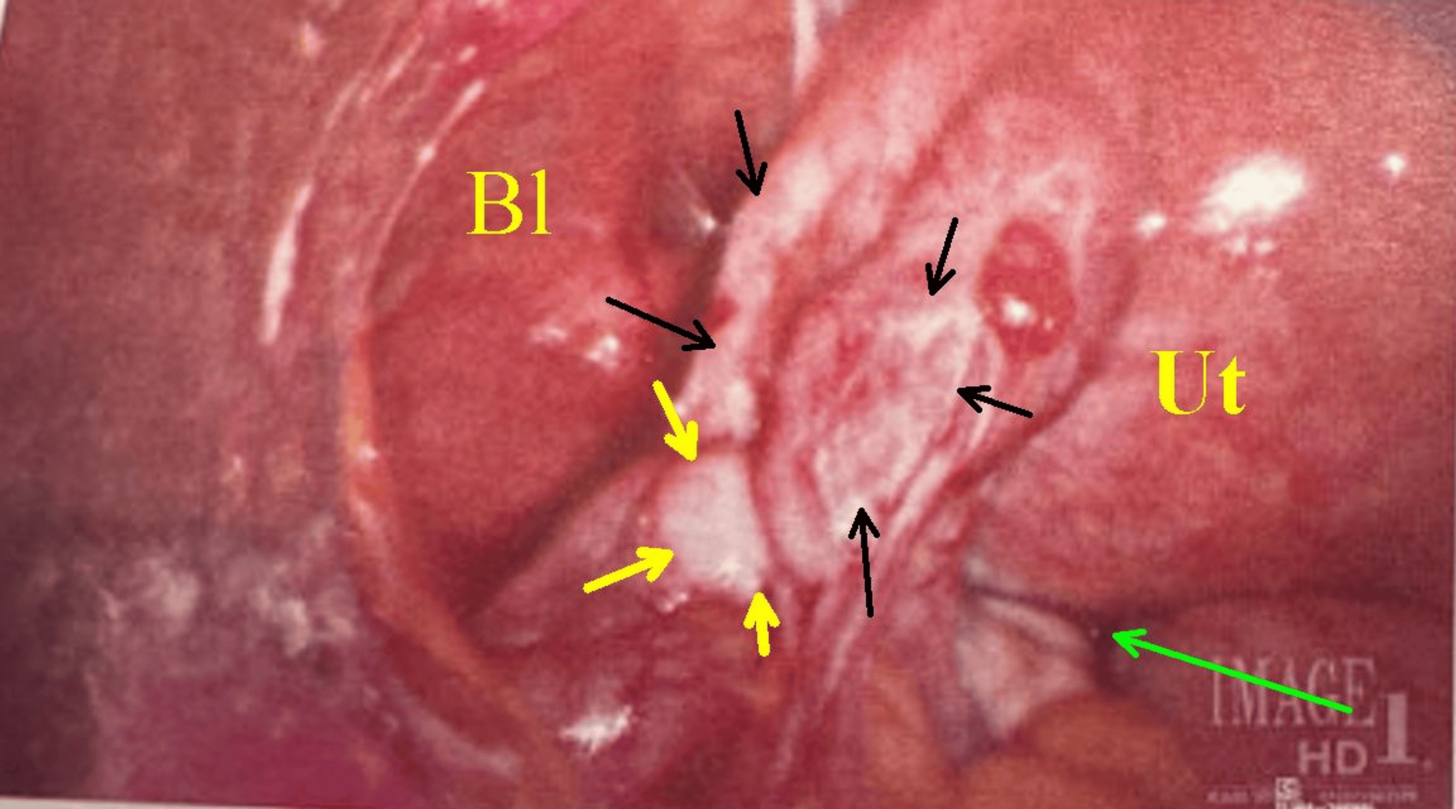
Laparoscopic view of the left pelvis. Bl is the bladder. Ut is the posterior uterine wall. The yellow arrows point to the surface of the left ovary; the black arrows encompass the inflammatory mass, including adhesions and tube in the mesosalpinx; and the green arrow is the rectouterine pouch.

After discussion and shared decision-making, the plan was adjusted to proceed with surgical management. The patient underwent diagnostic laparoscopy (Figure 7), bilateral salpingectomy, lysis of adhesions, and IUD removal approximately one week later.

**Figure 8:**
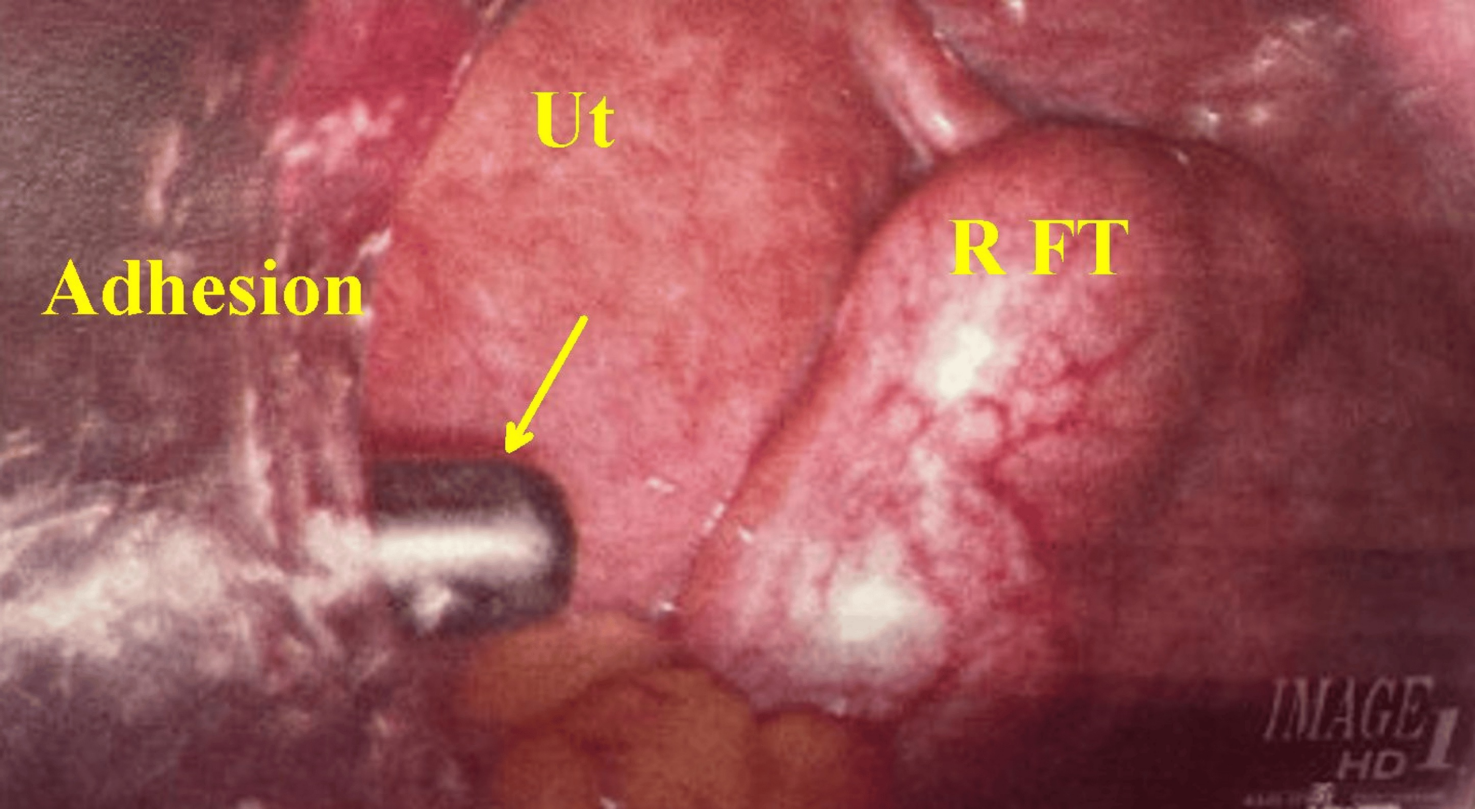
Close-up of the right pelvis through laparoscopy. The yellow arrow indicates the tip of the bipolar probe dissecting beneath a sheet of adhesions. Ut is the uterine wall. R FT shows the surface of the swollen right tubal inflammatory mass that had been documented in serial exams CTAP and TVUS. CTAP: computed tomography of the abdomen and pelvis, TVUS: transvaginal ultrasound.

Intraoperative findings included edematous bilateral fallopian tubes, with the right pyosalpinx (Figure 8) containing purulent fluid spilling into the pelvis. Intraoperative cultures were not collected. Additionally, pelvic adhesions were released, and a thorough intraoperative washout was performed. She was discharged on amoxicillin-clavulanate for one week. At her postoperative follow-up, she reported completing the antibiotics and experiencing a resolution of her pain.

## Discussion

TOA is a serious complication of PID, characterized by an inflammatory mass involving the fallopian tube, ovary, and sometimes nearby pelvic organs. While treatment with broad-spectrum antibiotics is the first choice, up to 25% of TOAs do not respond and need surgical or IR-guided drainage [1]. Despite clinical improvement, the TOA remained undrained due to perceived procedural risk by IR, delaying her recovery. Additionally, the patient had an IUD in place but consistently refused removal, requesting that it not be removed until she received permanent sterilization. This case highlights the diagnostic and therapeutic challenges in managing a recurrent, complex TOA complicated by bacteremia and an IUD with possible malposition.

Initial management with ceftriaxone, metronidazole, and doxycycline was appropriate and aligned with the CDC guidelines for severe PID and TOA [3]. Failed therapy is generally defined as new or persistent fever, ongoing or worsening pain, leukocytosis, enlarging mass, or suspected sepsis after 48-72 hours. The patient did not meet these criteria initially. However, her intermittent fevers and worsening respiratory symptoms required additional supportive care, emphasizing the systemic inflammatory burden a TOA can cause, even without frank rupture. Her clinical course became more concerning with the development of hypoxia, chest pain, and radiographic evidence of dependent pleural effusions, suggestive of a systemic inflammatory response, possibly related to peritoneal inflammation, early abscess rupture, and bacteremia. Transaminitis that arose during her initial hospitalization could have also indicated inflammation from abscess leakage rather than being solely attributed to ceftriaxone.

The recurrence of symptoms, combined with cervical motion tenderness, raised concerns for ongoing infection. Surgical intervention was ultimately necessary and, in retrospect, should have been considered earlier in her clinical course. A lab value that could have helped anticipate and identify treatment failure is C-reactive protein (CRP), but it was not collected throughout this patient’s disease course. A retrospective study showed that patients received antibiotic therapy with or without image-guided drainage, with both groups displaying similar CRP levels at admission. The group that underwent drainage experienced a significantly greater reduction in CRP levels by day 4. A decrease of less than 37.1% in CRP was associated with a higher likelihood of treatment failure [4].

There is one reported case discussing *E. coli* bacteremia in the context of TOA with eventual rupture and sepsis. This was a complication of a scheduled hysteroscopy, dilation, and curettage, but it was notable for the absence of leukocytosis in an immunocompetent individual [5]. Our patient initially exhibited leukocytosis, but this resolved within five days. Despite ongoing symptoms, her CBC was normal prior to surgery. PID and TOA are often polymicrobial, but *E. coli* is among the most frequently isolated organisms, along with aerobic *Streptococcus*, *Bacteroides *species, *Peptostreptococcus*, and *Peptococcus *[6]. The presence of purulent fluid and extensive adhesions observed during diagnostic laparoscopy, along with evidence of salpingitis, supports the likelihood of TOA rupture or leakage prior to surgery, even though she showed no obvious symptoms of peritonitis or sepsis. Bilateral salpingectomy and removal of the IUD resulted in symptom resolution and probably decreased the risk of future recurrence. Delayed surgical intervention is common in TOA cases where initial medical therapy provides only a partial response and does not fully eradicate the infection [1].

The key inflection point in this case was probably the persistence of the adnexal mass despite normalized labs, and these findings should prompt reconsideration of conservative management. Normalization of laboratory values may be misleading in deep-seated infections such as TOA. Also, the inclusion of missing serum CRP and intraoperative cultures could have improved her ongoing analysis and prognosis.

This case is notable for three reasons. The timing of an IUD insertion and the onset of active pelvic infection is probably not coincidental. A history of PID is generally considered a contraindication to IUD insertion unless a subsequent successful intrauterine pregnancy has occurred [7] and her IUD was inserted just a year before presenting with her active pelvic infection. Secondly, this case underscores the importance of multidisciplinary collaboration in managing complex TOA and timely re-evaluation for surgical intervention when clinical progress stalls. Finally, it highlights the need for close outpatient follow-up, given the high risk of recurrence and potential for complications.

## Conclusions

A TOA complicated by *E. coli* bacteremia and suspected rupture presents a rare but serious clinical scenario. This case underscores the importance of early recognition of systemic infection signs, prudent use of imaging and culture data, and prompt surgical intervention when conservative management fails. A high index of suspicion for rupture should be maintained in any patient with persistent symptoms, bacteremia, or systemic deterioration despite appropriate antibiotic therapy, even if laboratory findings are normal.
